# Chemotherapy Treatment of Elderly Patients (≥70 Years) with Non-Small Cell Lung Cancer: A Seven-Year Retrospective Study of Real-Life Clinical Practice at Karolinska University Hospital, Sweden

**DOI:** 10.1155/2015/317868

**Published:** 2015-07-14

**Authors:** Hirsh Koyi, Gunnar Hillerdal, Olov Andersson, Karl-Gustav Kölbeck, Per Liv, Eva Brandén

**Affiliations:** ^1^Department of Respiratory Medicine, Gävle Hospital, 80187 Gävle, Sweden; ^2^Karolinska Institutet, Stockholm, Sweden; ^3^Centre for Research and Development, Uppsala University, County Council of Gävleborg, Gävle, Sweden; ^4^Department of Respiratory Medicine and Allergy, Karolinska University Hospital, Solna, Stockholm, Sweden

## Abstract

An increasing proportion of cancer patients are aged >65 years and many are aged >70 years. Treatment of the elderly with lung cancer has, therefore, become an important issue; so we performed a retrospective study of our patients to demonstrate how elderly patients with NSCLC are treated in real-life, clinical practice. All patients aged ≥70 years with NSCLC at our department were reviewed retrospectively. In total, 1059 patients (50.8% of all NSCLC patients). Of these patients, 243 (22.9%) received chemotherapy, 164 (70.4%) of whom were treated with a platinum doublet using carboplatin. Second- and third-line chemotherapy were given to 31.4% and 13.9% of patients, respectively. Median overall survival was 289 and 320 days for male and female patients, respectively. Patients with performance status (PS) 0 experienced significantly better survival than patients with PS1 or PS 2: 410, 314, and 204 days, respectively. Age was of less importance, with patients aged 70–79 years versus those aged ≥80 years. Treatment of elderly NSCLC patients with chemotherapy is feasible if they have a good PS and appears to prolong survival. In this study, we found no significant differences in survival either between age groups or genders.

## 1. Introduction

Lung cancer (LC) is one of the most important causes of cancer morbidity and mortality worldwide [1]. In Sweden, LC is the number one cause of cancer mortality. Due to a general increase in life expectancy in most countries, the proportion of elderly in the general population is steadily growing, causing new social and health problems. The risk of cancer also increases with age [2]; consequently, the number of patients with LC aged >65 years is increasing. This trend is expected to continue over the next decades [3] and the proportion of the population aged >70 years is expected to double by 2020 [4].

Currently, over 50% of LC patients are aged >65 years and 30–40% are aged >70 years [5, 6], whilst in Sweden, the median age at diagnosis is 71 years. In a recent community hospital-based survey in France, these figures were lower; only 32% of LC patients were aged ≥70 years and 18% were aged >80 years [7]. Conversely, in the US, patients aged >65 years represented two-thirds of LC cases and the median age at diagnosis was almost 70 years [3].

Non-small cell lung cancer (NSCLC) accounts for 80–85% of all LC types. At time of diagnosis, the tumour is often advanced, leaving only systemic palliative chemotherapy or molecular targeted therapy in those who are fit to choose from as possible therapeutic options. Furthermore, elderly patients often suffer from extensive comorbidity, making treatment more challenging. Thus, despite the fact that patients aged >70 years also derive a clinical benefit from systemic chemotherapy [8, 9], they are at risk of being undertreated [10, 11]. Notably, no consensus definition of “elderly” exists [12]. Some investigators use 65 years as a cut-off point, but the majority of studies specify 70 years, having been described as the lower limit of senescence, that is, the age at which the incidence of comorbidity increases sharply [13].

The purpose of this study was to describe chemotherapy treatment provided to elderly patients with NSCLC in clinical practice in our hospital and to assess their chemotherapy tolerance and survival. We also sought to identify the reasons for discontinuation of planned chemotherapy. In the absence of clear evidence from clinical trials, analysis of the elderly NSCLC population through retrospective population-based studies, such as this study, helps physicians to assess and quantify the value of treating elderly cancer patients. Since diagnostic work-up and treatment of LC in the northern part of Stockholm is centralized to the Solna site of Karolinska University Hospital, this study included practically all patients in a population base of 1.1 million inhabitants.

## 2. Material and Methods

This was a single-centre, retrospective cohort study in all elderly patients (aged ≥70 years) diagnosed in the Department of Respiratory Medicine and Allergy at the Karolinska University Hospital in Solna, Stockholm, from 1st January, 2003, to 31st December, 2009.

Patients were identified using the Stockholm-Gotland Cancer Registry, which is known for its high level of comprehensive data reporting.

The following variables were included in the retrospective analysis: gender, smoking history, performance status (PS) according to WHO, comorbidities, Charlson Comorbidity Index (CCI), TNM stages [14], LC type, total numbers of lines of treatment, type of chemotherapy given and overall survival, whether the patient received all chemotherapy cycles, number of cycles received, and reasons for discontinuing chemotherapy. A complete course of chemotherapy was defined as the patient having received any of the regimens once every three weeks for four cycles. Information about other treatments patients had received, such as second- or third-course treatment, was also collected. The last study observation was made on 18th November, 2014.

A multidisciplinary meeting, always attended by oncologists, pulmonologists, a radiologist, a pathologist, and LC nurses, was held once a week at the hospital, during which all LC cases were discussed and determined the course of treatment in LC patients based on Swedish LC guidelines. Notably, almost all patients with suspected LC are referred to hospital pulmonary departments in Sweden.

The study was approved by the Ethical Committee of the Karolinska Institutet 2005/115.

## 3. Statistics

Statistics software SPSS (version 22.0) was used for all study analyses. Patient characteristics at point of diagnosis were summarized using standard descriptive statistics. Observed frequencies in categorical variables were calculated. Survival time after diagnosis was analysed using Kaplan-Meier estimates, and differences in survival distributions for different patient subgroups were tested using log-rank tests. Differences in categorical variables between subgroups were statistically tested using Fishers exact test, using Monte Carlo simulation with 20 000 replicates for variables with more than two categories. The significance level was set at 0.05 for all statistical tests. Comorbidities were analysed according to CCI [15].

## 4. Results

In total, 2351 patients were newly diagnosed with LC during the period 2003–09 in this department. Of these, 267 (11.3%) had small cell lung cancer and were excluded from this study, leaving 2084 patients with NSCLC in the study. Of these 2084 patients, 1059 patients (50.8% of all NSCLC patients) were aged ≥70 years.

Of the 1059 elderly patients with NSCLC, 243 (22.9%) received chemotherapy; 49 patients were aged ≥80 years (almost 20% of the elderly, treated patients). There were 126 (51.9%) males with a mean age of 75.8 years (median 75.5, range 70–86) and 117 (48.1%) females, also with a mean age of 75.8 years (median 75.0, range 70–89).

Of the treated male patients, 30.2% were smokers, 61.9% were ex-smokers, and 7.9% had never smoked; corresponding figures for female patients were 35.9%, 43.6%, and 20.5%, respectively. The difference in smoking habits between genders was statistically significant (*p* = 0.003) (Table 1).

Adenocarcinoma was the most prevalent histology type and occurred in 136 (56.0%) patients; squamous cell carcinoma was the second most prevalent and occurred in 63 (25.9%) patients. Low differentiated NSCLC occurred in 30 (12.3%) patients. A histological diagnosis was not available in 3 (1.2%) patients for ethical reasons. There was no statistically significant gender difference in histology types (Table 1).

Of those treated, 44 patients (18.1%) had a performance status (PS) of 0, 140 (57.6%) PS 1, and 59 (24.3%) PS 2. There was no statistical significant difference in PS (*p* = 0.8). Stage IV was the most common LC stage, seen in 131 patients (53.9%); stages IIIb and IIIa were found in 75 (30.9%) and 28 (11.5%) patients, respectively. There were no significant gender differences in LC staging (*p* = 0.3) (Table 1).

Of the chemotherapy treated patients, 182 (73.8%) received carboplatin plus gemcitabine and a further 6 (3.5%) patients received another platinum combination. In total, therefore, all 77.3% of patients were treated with a platinum doublet, 17 (7.3%) patients received gemcitabine single, and 17 (7.3%) received vinorelbine single (Table 2). Most patients (115, 49.9%) received at least four cycles and another 37 (15.9%) patients received three cycles.

**Figure 1:**
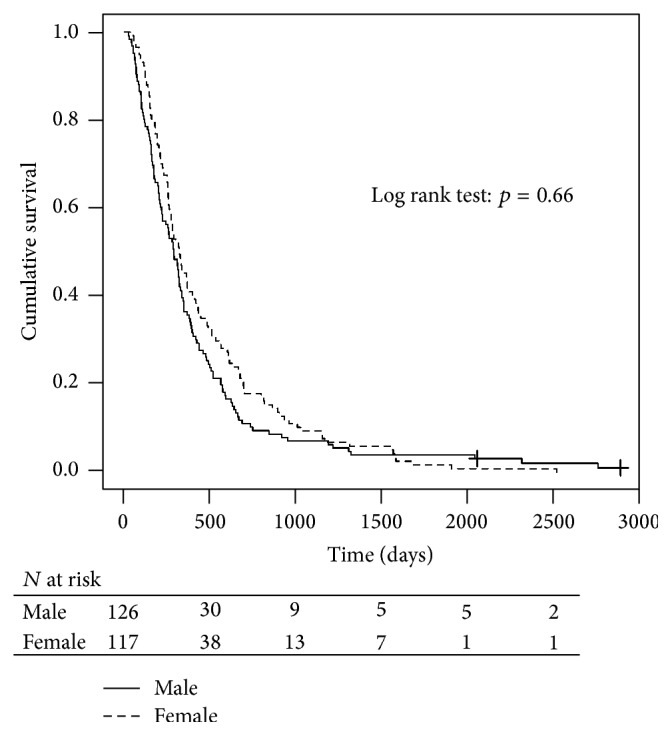
Kaplan-Meier estimates of overall survival between the genders.

Chemotherapy was discontinued in 30 patients (13.1%) due to nonhaematological toxicities, in 35 (15.3%) due to haematological toxicity, and in 38 (16.6%) due to disease progression or death (Table 3). In total, therefore, 121 patients (52.8%) completed their treatment. The median overall survival for the whole, treated group was 308 days with no significant gender differences (*p* = 0.414) (Figure 1).

Second- and third-line chemotherapy were given to 76 (31.4%) patients and third-line chemotherapy was given to 33 (13.9%) patients.

**Figure 2:**
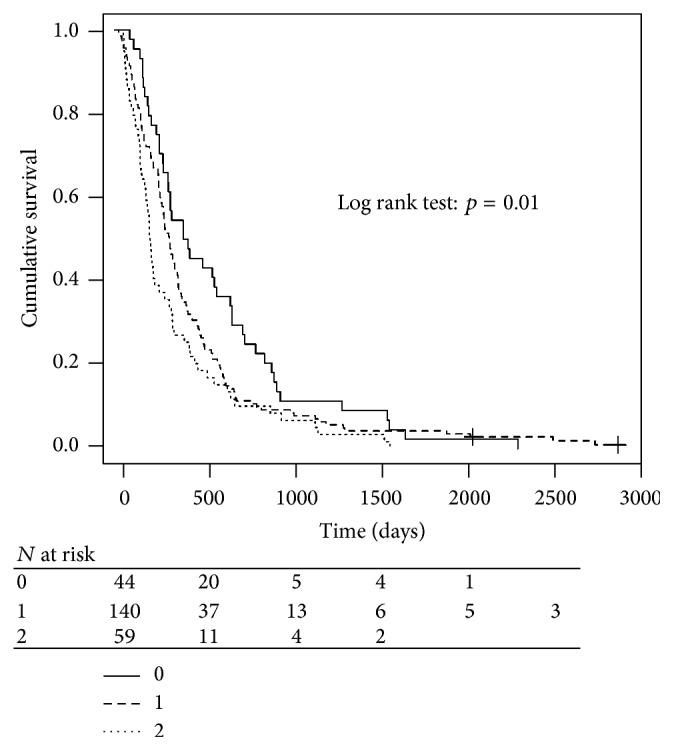
Overall survival by performance status.

**Figure 3:**
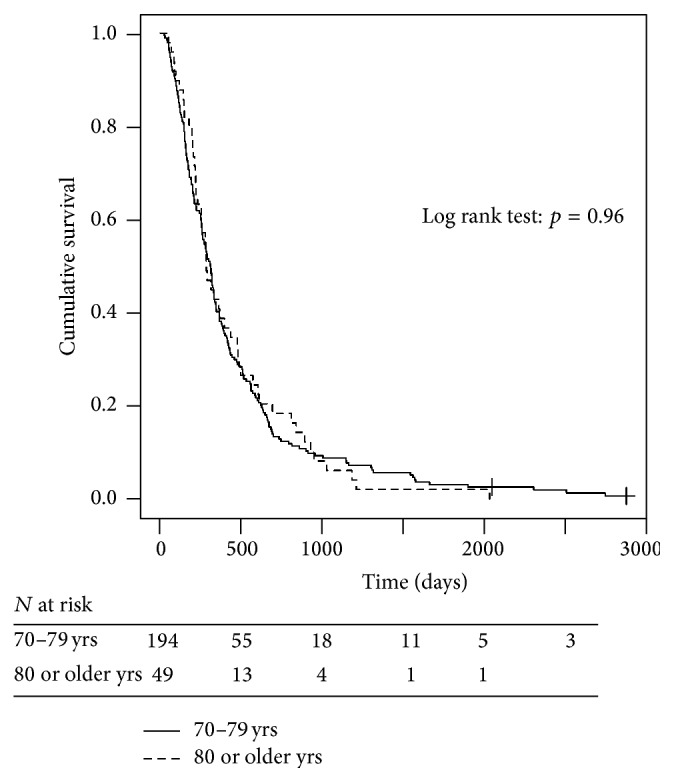
Overall survival by age group.

Both age and PS influenced survival, but PS was of greater importance. Survival among patients with PS 0 was 410 days, was 314 days in those with PS 1, and was 204 days in those with PS 2. There was a significant survival difference between PS 0 and both PS 1 (*p* = 0.084) and PS 2 (*p* = 0.003) (Figure 2). There was also a significant survival difference between PS 1 and PS 2 (*p* = 0.055). The median survival was 310 days in patients aged 70–79 years and 285 days in patients aged ≥80 years (*p* = 0.963) (Figure 3). The CCI had no effect on survival, with a median survival in CCI 0, 1, 2, and 3 of 283, 324, 270, and 262 days, respectively (*p* = 0.663). There was no significant difference in overall median survival during the periods 2003–06 and 2007–09 (286 and 320 days, resp., *p* = 0.657).

## 5. Discussion

This is, to our knowledge, the first study to comprehensively evaluate chemotherapy treatment in elderly patients with NSCLC in real-life, clinical practice. This study demonstrates that more than 50% of the NSCLC patients diagnosed at our department were aged ≥70 years; yet only about 25% of them received chemotherapy. Elderly patients require an additional degree of consideration because of the increasing prevalence of comorbidities and reduction in physiological reserve, which may influence the possibility of administering chemotherapy safely, especially doublet regimens [8, 9]. Little data exist, however, regarding the outcome of chemotherapy in NSCLC patients aged ≥80 years, which is a rapidly expanding, potentially vulnerable population cohort. Most standard definitions for “frailty” in geriatric texts define people aged >85 years as frail; by definition, this group has insufficient physiological reserve to tolerate standard chemotherapy [16]. Hesketh and colleagues [17] analysed the effect of either sequential vinorelbine followed by docetaxel or weekly, single agent docetaxel in the 21.5% of patients aged >80 years in their elderly-specific studies. Compared to the younger group (aged <80 years), the response rate was lower (8% versus 17%), as was the overall survival in the more elderly group when patients with poor PS were excluded (7 versus 11 months). Severe toxicities were similar [17]. In our study, 20.2% of the patients were aged ≥80 years. The survival of patients who were treated was 310 days in those aged 70–79 years and 285 days in patients aged ≥80 years (*p* = 0.9).

Treatment guidelines for stage IV NSCLC of the American College of Chest Physicians recommend chemotherapy for patients aged 70–79 years (Grade 1A) and for select patients aged ≥80 years on a case by case basis (Grade 2C) [18]. A number of prospective trials in advanced elderly NSCLC patients have established clearly the efficacy of single agent chemotherapy compared to best supportive care (BSC) in patients aged ≥70 years [19–21]. In a Phase III trial specifically designed for elderly LC patients in France, carboplatin and paclitaxel both conferred significant improvement in overall survival (OS) compared with single agent vinorelbine or gemcitabine [22].

The standard first-line treatment for fit, elderly patients with advanced NSCLC is, therefore, chemotherapy. Unfortunately, the proportion of elderly patients receiving this treatment is still low [23–25], primarily due to concern about drug-related, toxic side effects [26]. A survey from the SEER showed that only a minority of patients aged >65 years received chemotherapy for an advanced NSCLC (25.8% of 21.2% patients aged ≥66 years), while in France, 61.5% of 1627 patients aged ≥70 years received chemotherapy [27].

Response rates and survival are reported to be lower in the more elderly population despite similar toxicities [3, 23, 28]. Age* per se* is not the determinant factor of poor prognosis in advanced cancer [29–31] but rather the physiological status, daily living capacity, and other factors [32]. This is consistent with our study, which showed PS to be more important than age as regards prognosis.

The role of platinum-based regimens in the elderly remains controversial. A retrospective analysis of cisplatin-based chemotherapy in NSCLC patients revealed a significant increase in mortality within 30 days of starting chemotherapy with increasing age [33], while other studies have shown good tolerance and benefit even in patients aged ≥80 years [34]. Schedule and dose of chemotherapy treatments in the elderly both require further investigation; consequently, special care is required when using cisplatin-based therapy in elderly patients [3]. In our study, most patients received carboplatin-based chemotherapy because this is probably less nephrotoxic than cisplatin.

Generally, it is accepted that NSCLC patients who either are female or have never smoked live longer than men [33, 35, 36]. This was not, however, observed in elderly patients in this study. The influence of gender and smoking status was very limited in our patients.

In conclusion, our retrospective study supports the concept that chemotherapy that includes platinum is feasible in elderly LC patients. The use of carboplatin appears to be more suitable for this population, with less toxicity than cisplatin, and can be used in association with new drugs in fit, elderly patients with good PS. Larger, prospective studies in elderly patients with advanced-stage NSCLC are needed to both guide clinical management and therapeutic decisions and improve treatment outcomes in such patients.

## Figures and Tables

**Table 1:** Patient characteristics.

	Male	Female	Total
Patients *N* (%)	126 (51.9%)	117 (48.1%)	243 (100%)
Age			
Mean	75.8	75.8	
Median	75.5	75	
Range	70–86	70–89	
Smoking^a^			
Current smoker	38 (30.2%)	42 (35.9%)	80 (32.9%)
Former smoker	78 (61.9%)	51 (43.6%)	129 (53.1%)
Never smoked	10 (7.9%)	24 (20.5%)	34 (14.0%)
PS (WHO)^b^			
0	24 (19.0%)	20 (17.1%)	44 (18.1%)
1	73 (57.9%)	67 (57.3%)	140 (55.6%)
2	29 (23.0%)	30 (25.6%)	59 (24.3%)
Type of lung cancer^c^			
Adenocarcinoma	66 (52.4%)	70 (59.8%)	136 (56.0%)
Squamous cell carcinoma	39 (31.0%)	24 (20.5%)	63 (25.9%)
Low differentiated cell carcinoma	15 (11.9%)	15 (12.8%)	30 (12.3%)
Lung cancer, clinical diagnosis	1 (0.8%)	2 (1.7%)	3 (1.2%)
Large cell carcinoma	5 (4.0%)	5 (4.3%)	10 (4.1%)
Adenosquamous cell carcinoma	0 (0.0%)	1 (0.9%)	1 (0.4%)
Total	126 (51.8%)	117 (48.2%)	243 (100.0%)
Tumour stage^d^			
Ia	1 (0.8%)	0 (0.0%)	1 (0.4%)
Ib	1 (0.8%)	3 (2.6%)	4 (1.6%)
IIa	1 (0.8%)	0 (0.0%)	1 (0.4%)
IIb	0 (0.0%)	3 (2.6%)	3 (1.2%)
IIIa	14 (11.1%)	14 (12.0%)	28 (11.5%)
IIIb	42 (33.3%)	33 (28.2%)	75 (30.9%)
IV	67 (53.2%)	64 (54.7%)	131 (53.9%)
Total	126 (51.8%)	117 (48.2%)	243 (100.0%)

^a^ *p* = 0.00365; ^b^ *p* = 0.8585; ^c^ *p* = 0.4461; ^d^ *p* = 0.3752.

**Table 2:** Type of chemotherapy given to patients.

Type of chemotherapy	Number of patients (%)
Carboplatin-gemcitabine	182 (73.8%)
Gemcitabine	17 (7.3%)
Vinorelbine	17 (7.3%)
Paclitaxel-carboplatin	2 (0.9%)
Cisplatin-pemetrexed	4 (1.7%)
Paclitaxel	5 (2.1%)
Gemcitabine-pemetrexed	15 (6.4%)
Pemetrexed	1 (0.4%)
Total	243 (100.0%)

**Table 3:** Reasons for discontinuation of chemotherapy in patients.

Reason for chemotherapy discontinuation	Number of patients (%)
Died during treatment	30 (13.1%)
Nonhaematological toxicities	30 (13.1%)
Haematological toxicities	35 (15.3%)
Disease progress	8 (3.5%)
Patient's preference	6 (2.2%)

## References

[1] Ferlay J., Steliarova-Foucher E., Lortet-Tieulent J. (2013). Cancer incidence and mortality patterns in Europe: estimates for 40 countries in 2012. *European Journal of Cancer*.

[2] Mistry M., Parkin D. M., Ahmad A. S., Sasieni P. (2011). Cancer incidence in the United Kingdom: projections to the year 2030. *British Journal of Cancer*.

[3] Owonikoko T. K., Ragin C. C., Belani C. P. (2007). Lung cancer in elderly patients: an analysis of the surveillance, epidemiology, and end results database. *Journal of Clinical Oncology*.

[4] Hoffe S., Balducci L. (2012). Cancer and age: general considerations. *Clinics in Geriatric Medicine*.

[5] Weir H. K., Thompson T. D., Soman A., Møller B., Leadbetter S. (2015). The past, present, and future of cancer incidence in the United States: 1975 through 2020. *Cancer*.

[6] Gridelli C., Balducci L., Ciardiello F. (2015). Treatment of elderly patients with non-small cell lung cancer: results of an International Expert Panel Meeting of the Italian Association of Thoracic Oncology. *Clinical Lung Cancer*.

[7] Piquet J., Blanchon F., Grivaux M. (2003). Primary bronchial carcinoma in elderly subjects in France. *Revue des Maladies Respiratoires*.

[8] The Elderly Lung Cancer Vinorelbine Italian Study Group (1999). Effects of vinorelbine on quality of life and survival of elderly patients with advanced non-small-cell lung cancer. *Journal of the National Cancer Institute*.

[9] Gridelli C., Perrone F., Gallo C. (2003). Chemotherapy for elderly patients with advanced non small-cell lung cancer: the Multicenter Italian Lung Cancer in the Elderly Study (MILES) phase III randomized trial. *Journal of the National Cancer Institute*.

[10] Denson A. C., Mahipal A. (2014). Participation of the elderly population in clinical trials: barriers and solutions. *Cancer Control*.

[11] Lewis J. H., Kilgore M. L., Goldman D. P. (2003). Participation of patients 65 years of age or older in cancer clinical trials. *Journal of Clinical Oncology*.

[12] Hotta K., Ueoka H., Kiura K., Tabata M., Tanimoto M. (2004). An overview of 48 elderly-specific clinical trials of systemic chemotherapy for advanced non-small cell lung cancer. *Lung Cancer*.

[13] Gridelli C., Langer C., Maione P., Rossi A., Schild S. E. (2007). Lung cancer in the elderly. *Journal of Clinical Oncology*.

[14] Mountain C. F. (1986). A new international staging system for lung cancer. *Chest*.

[15] Charlson M. E., Pompei P., Ales K. L., MacKenzie C. R. (1987). A new method of classifying prognostic comorbidity in longitudinal studies: development and validation. *Journal of Chronic Diseases*.

[16] Balducci L. (2003). Geriatric oncology. *Critical Reviews in Oncology/Hematology*.

[17] Hesketh P. J., Lilenbaum R., Chansky K. (2005). Chemotherapy in patients ≥ 80 with advanced non-small cell lung cancer. Combined results from SWOG 0027 and LUN 6. *Journal of Clinical Oncology*.

[18] Alberts W. M. (2007). American College of Chest Physicians. Diagnosis and management of lung cancer executive summary: ACCP evidence-based clinical practice guidelines (2nd Edition). *Chest*.

[19] Frasci G., Lorusso V., Panza N. (2000). Gemcitabine plus vinorelbine versus vinorelbine alone in elderly patients with advanced non-small-cell lung cancer. *Journal of Clinical Oncology*.

[20] Gridelli C., Perrone F., Gallo C. (2003). Chemotherapy for elderly patients with advanced non-small-cell lung cancer: the multicenter Italian lung cancer in the elderly study (MILES) phase III randomized trial. *Journal of the National Cancer Institute*.

[21] Kudoh S., Takeda K., Nakagawa K. (2006). Phase III study of docetaxel compared with vinorelbine in elderly patients with advanced non-small-cell lung cancer: results of the West Japan Thoracic Oncology Group trial (WJTOG 9904). *Journal of Clinical Oncology*.

[22] Quoix E. A., Oster J., Westeel V. (2010). Weekly paclitaxel combined with monthly carboplatin versus single-agent therapy in patients age 70 to 89: IFCT-0501 randomized phase III study in advanced non-small cell lung cancer (NSCLC). *Journal of Clinical Oncology*.

[23] Schiller J. H., Harrington D., Belani C. P. (2002). Comparison of four chemotherapy regimens for advanced non-small-cell lung cancer. *The New England Journal of Medicine*.

[24] Davidoff A. J., Tang M., Seal B., Edelman M. J. (2010). Chemotherapy and survival benefit in elderly patients with advanced non-small-cell lung cancer. *Journal of Clinical Oncology*.

[25] Davidoff A. J., Tang M., Seal B., Edelman M. J. (2010). Chemotherapy and survival benefit in elderly patients with advanced non-small-cell lung cancer. *The Cancer Journal*.

[26] Hutchins L. F., Unger J. M., Crowley J. J., Coltman C. A., Albain K. S. (1999). Underrepresentation of patients 65 years of age or older in cancer-treatment trials. *The New England Journal of Medicine*.

[27] Quoix E., Monnet I., Scheid P. (2010). Management and outcome of French elderly patients with lung cancer: an IFCT survey. *Revue des Maladies Respiratoires*.

[28] Hesketh P. J., Lilenbaum R. C., Chansky K. (2007). Chemotherapy in patients ≥80 with advanced non-small cell lung cancer: combined results from SWOG 0027 and LUN 6. *Journal of Thoracic Oncology*.

[29] Albain K. S., Crowley J. J., LeBlanc M., Livingston R. B. (1991). Survival determinants in extensive-stage non-small-cell lung cancer: the southwest oncology group experience. *Journal of Clinical Oncology*.

[30] Clement D., Miron L., Marinca M. (2007). Age-related prognostic factors and treatment results for advanced non-small cell lung cancer (NSCLC). *Revista Medico-Chirurgicala a Societatii de Medici si Naturalisti din Iasi*.

[31] Ansari R. H., Socinski M. A., Edelman M. J. (2011). A retrospective analysis of outcomes by age in a three-arm phase III trial of gemcitabine in combination with carboplatin or paclitaxel vs. paclitaxel plus carboplatin for advanced non-small cell lung cancer. *Critical Reviews in Oncology/Hematology*.

[32] Quoix E., Zalcman G., Oster J.-P. (2011). Carboplatin and weekly paclitaxel doublet chemotherapy compared with monotherapy in elderly patients with advanced non-small-cell lung cancer: IFCT-0501 randomised, phase 3 trial. *The Lancet*.

[33] Asmis T. R., Ding K., Seymour L. (2008). Age and comorbidity as independent prognostic factors in the treatment of non-small-cell lung cancer: a review of National Cancer Institute of Canada Clinical Trials Group Trials. *Journal of Clinical Oncology*.

[34] Altundag O., Stewart D. J., Fossella F. V. (2007). Many patients 80 years and older with advanced non-small cell lung cancer (NSCLC) can tolerate chemotherapy. *Journal of Thoracic Oncology*.

[35] Kawaguchi T., Takada M., Kubo A. (2010). Gender, histology, and time of diagnosis are important factors for prognosis: analysis of 1499 never-smokers with advanced non-small cell lung cancer in japan. *Journal of Thoracic Oncology*.

[36] Sun S., Schiller J. H., Gazdar A. F. (2007). Lung cancer in never smokers—a different disease. *Nature Reviews Cancer*.

